# Differentiation of Testis Xenografts in the Prepubertal Marmoset Depends on the Sex and Status of the Mouse Host

**DOI:** 10.3389/fendo.2018.00467

**Published:** 2018-08-29

**Authors:** Swati Sharma, Reinhild Sandhowe-Klaverkamp, Stefan Schlatt

**Affiliations:** Center of Reproductive Medicine and Andrology, Institute of Reproductive and Regenerative Biology, Münster, Germany

**Keywords:** endocrine, sex, testis, xenograft, marmoset, spermatogenesis, castration, prepubertal

## Abstract

This study investigates the effects of the endocrine milieu of immunodeficient mouse host (intact vs. castrated male, intact male vs. intact female) on prepubertal marmoset (*Callithrix jacchus*) testicular xenografts. Previous marmoset xenografting studies used castrated nude mouse hosts which did not support efficient graft survival and maturation. Due to the distinct endocrine milieu in marmosets with a deletion of exon 10 in the LH receptor, we wanted to explore whether the most efficient xenograft development occurs in intact male mouse hosts compared to intact females or castrated males. We xenografted freshly isolated tissue from prepubertal marmosets (age range 4–6 months) into the back skin of three groups of nude mice (intact male, castrated male, and intact female). We collected serum for endocrine determinations and grafts after 20 weeks and determined hormonal/reproductive status, graft survival, somatic cell development and initiation of germ cell differentiation. Graft development, tubular integrity, and germ cell differentiation status in the grafts retrieved from different hosts was scored by morphometric analysis. The influence of the different endocrine status was compared between groups of hosts. Endocrine readouts and histological endpoints in xenografts substantiate that grafts were exposed to different microenvironments and responded with host specific developmental patterns. The intact male hosts supported the most significant progression of germ cell development. Our data provide evidence for the important role of the host milieu on survival and differentiation of marmoset xenografts. The xenografting model offers innovative avenues to exploit development and endocrine effects in the primate marmoset testis using limited numbers of non-human primates for the experimental settings.

## Introduction

Xenografting of testicular tissue can be considered co-transplantation of germ cells with its somatic microenvironment into a host. Meiotic differentiation in xenografted fragments is supported by complete restoration of vascular supply from the host which provides optimal nutrient and oxygen supply, facilitating spermatogenic and androgenic induction in testicular fragments (1, 2). The subcutaneous transplantation technique has been successfully used as an experimental tool to generate sperm from testicular fragments of various species (mouse, pig, goat, sheep, hamster, bull, rhesus monkey) (1, 3–9). Regarding the potential clinical applications pre-pubertal male cancer patients undergoing chemotherapy are exposed to high dose gonadotoxic treatment depleting spermatogonial stem cells in their testes, rendering many young cancer survivors eventually infertile for life. The study gains relevance from the fact that ectopic xenografting of human prepubertal testicular tissue is currently being developed as a clinically viable fertility preservation strategy. However, an optimized strategy for host environment has yet to be established (10–15). Experimental exploration using non-human primates revealed the generation of functional sperm in ectopically grafted macaque testicular xenografts (1, 5, 8, 9, 16–18). Past marmoset and human xenografting studies investigated the effect of donor tissue (maturation status), effect of gonadotropins as well as time post grafting and site of grafting on xenograft survival and maturation (1, 12, 16, 19–22).

Previous studies investigating host effect show conflicting evidence. In several studies intact hosts were observed to efficiently support complete spermatogenesis (23–27). However, efficient xenograft survival, improved sperm production, reduced degeneration in castrated hosts were reported by others (14, 26). Castrated recipients were continued to be used as hosts, as absence of testis in the recipient allowed host gonadotropins to rise, further stimulating graft Leydig cells to produce androgens. Host castration at the time of grafting disrupted the negative feedback axis increasing FSH levels in the host. Higher gonadotropin levels in castrated recipients stimulated Sertoli cell proliferation and development of grafts until the HPG axis was re-established between the grafted tissue and the host pituitary (14).

New world monkeys are considered valuable preclinical models in male reproduction research. Common Marmosets (*Callithrix jacchus*) demonstrate comparable testicular development and function as observed in humans (28). This New World monkey has comparable epithelial arrangement, testicular physiology and function. Their spermatogonial progenitor system is similar to man rendering the marmoset testis a valid model for pre-clinical research to study spermatogenesis and to develop clinically viable fertility preservation strategies (29, 30, 32–35). Pubertal activation (high serum levels of testosterone) in marmosets initiates after 6 months of age (31). Asynchronous germ cell proliferation is observed during pre- and postnatal period in marmosets. Similar to man marmoset testis contains gonocytes until few weeks after birth (29) and undergoes testicular quiescence (30).

Previous marmoset xenografting studies in castrated hosts revealed, in comparison to other donor species, poor graft survival and spermatogenic induction. This was explained by the presence of a distinct endocrine system in marmosets. However, the aspect of primate tissue development and host effect was only marginally explored (1, 5, 16, 36–38). Marmosets have a mutated Luteinizing hormone (LH) receptor which lacks exon 10 making the LH receptor unresponsive to LH but responsive to CG (39–41). This mutation renders xenografted marmoset fragments non-responsive to endogenous LH released from the mouse pituitary (16), rendering this model specifically valid to observe FSH and steroid-dependent effects (39–43).

We aimed to describe the effect of host sex and status on marmoset graft survival and maturation. Preceding marmoset grafting studies, in contrast to many other species, revealed poor differentiation of testis xenografts in castrated hosts (1, 16, 36). We therefore postulated that a female or intact male host microenvironment affects marmoset graft development in different ways. In this study we explored whether graft survival, development and the degree of spermatogenic induction is dependent on the status of the host (intact vs. castrate, male vs. female).

## Materials and methods

### Ethical approval for animal use

Ethical approval for the use of marmosets (LANUV-NRW 8.87-50.10.46.09.018) and mice (LANUV-NRW 84-02.04.2012.A075) was obtained according to German federal law on the care and use of laboratory animals.

### Marmoset testicular donors and testicular tissue retrieval

Testes tissue from six prepubertal marmosets (*Callithrix jacchus*) bred at the institutional breeding facility, aged between 4 and 6 months were used for this study. All marmosets were housed in similar conditions in groups or families under a 12 h light/12 h photoperiod and were fed a diet containing fresh food and supplements, with food pellets (Altromin, Lage, Germany). They had unrestricted access to tap water. Before testes retrieval, the monkeys were anesthetized with ketaject (equivalent to 100 mg/ml ketamine per animal) and killed by decapitation. Body weights of all marmosets were recorded and blood was collected for serum testosterone analysis. All recorded parameters are shown in Table 1. Their total testicular weight ranged between 61 and 125 mg. All 12 testes retrieved from the 6 monkeys were de-capsulated, dissected and transferred to chilled Leibovitz-15 medium.

**Table 1:** Characterization of prepubertal marmoset monkey (*Callithrix jacchus*) donors.

***Callithrix jacchus***	**Maturity state**	**Age (years/months/days)**	**Body weight (g/kg)**	**Total testicular weight (mg/g)**
MC941	Pre-puberty	6 m 7 d	270 g	125.4 mg
MC945	Pre-puberty	6 m 4 d	263 g	77.8 mg
MC947	Pre-puberty	5 m 28 d	253 g	99.7 mg
MC959	Pre-puberty	4 m 25 d	248 g	72.5 mg
MC963	Pre-puberty	4 m 12 d	192 g	61.8 mg
MC964	Pre-puberty	4 m 12 d	175 g	68.9 mg

Prepubertal marmoset reference values: Age: 4–6 m, body weight: 184(±13) g, total testicular weight: 68.9–125.4 mg. Spg, Spermatogonia (43, 47)

### Preparation of testicular fragments for xenografting

**Figure 1:**
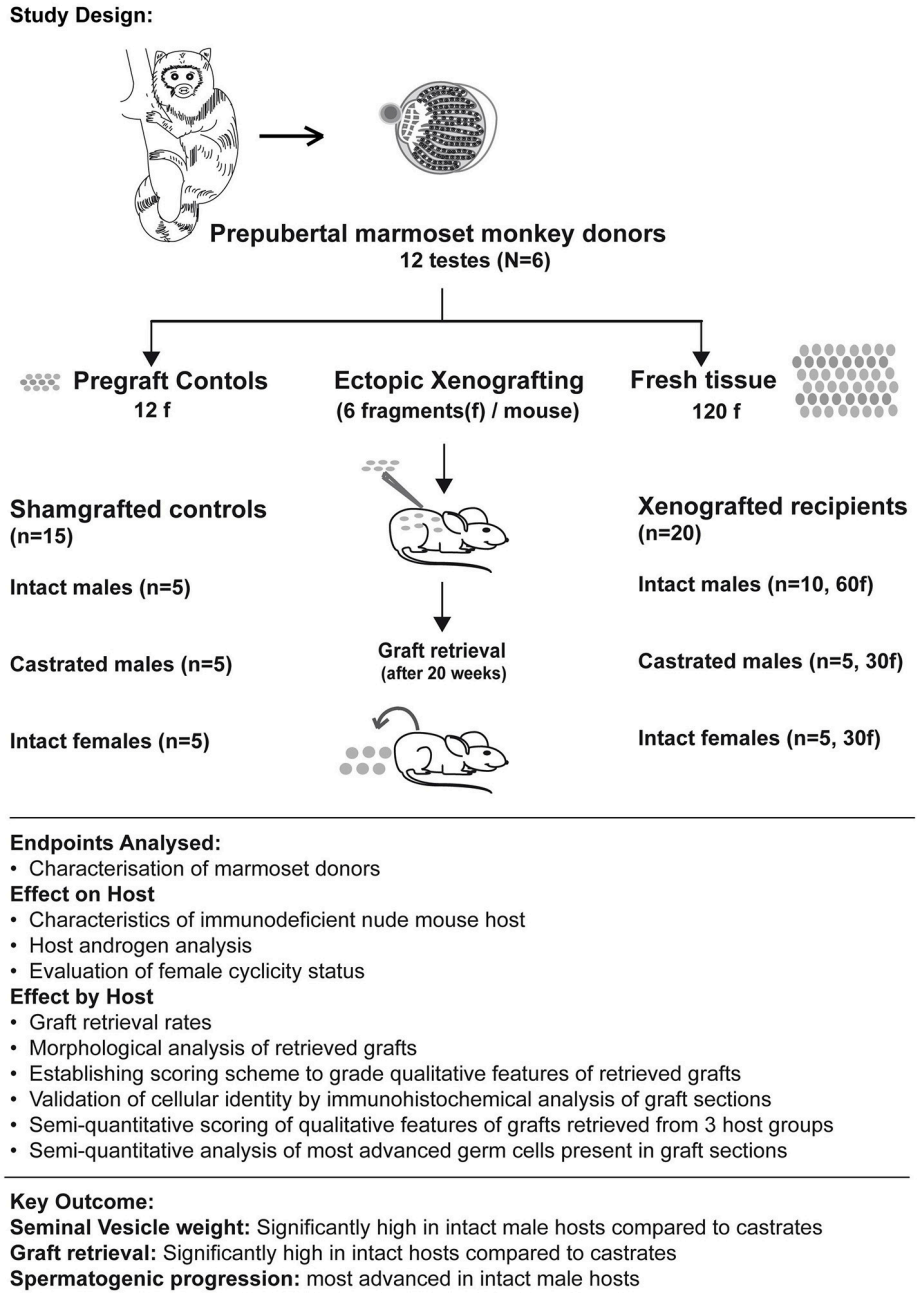
Summary figure illustrates the study design (including number of donor testes, hosts, and testicular fragments xenografted in each host group), endpoints analyzed (to evaluate effect of grafting on hosts and effect of hosts on graft survival and differentiation) and highlights the key outcome from the analysis.

Testicular tissue was dissected into 492 fragments (63–106 fragments per monkey) of uniform size (~1 mm3). The dissected tissue fragments were systematically divided into three groups. 12 fragments (one from each testis) were processed as pre-graft controls. These were fixed in Bouin's solution for 24 h, transferred into 70% ethanol and embedded into paraffin for sectioning prior to histological analysis. From all 6 marmoset donors, 20 testicular fragments were subjected to grafting. To avoid any bias and maintain homogeneity, these 120 testicular fragments were pooled before grafting. From this pool, fragments were randomly selected to be grafted into 20 mouse recipients receiving six grafts each (see Figure 1 and Table 2). Remaining fragments were stored on ice-cold Leibovitz-15 medium and cryopreserved using various methods for a parallel xenografting study investigating the cryopreservation of marmoset testicular tissue.

**Table 2:** Intact and castrated immunodeficient nude mouse host organ data.

**Host parameters**	**Intact males**		**Castrated males**		**Intact females**	
**Group**	**Sham**	**Grafted**	**Sham**	**Grafted**	**Sham**	**Grafted**
Grafted mice (survived)	4/5	7/10	3/5	4/5	5/5	5/5
Mean body weight (g)	40 ± 3	37.5 ± 3.2	38.5 ± 7	38 ± 1.4	36 ± 0.7	36 ± 1.2
Seminal vesicle weight (mg)	478 ± 92(AB)	372 ± 106(AB)	20.4 ± 18(a)	17 ± 2.3(b)	–	–
Testis weight (mg)	103.3 ± 2	107 ± 3	–	–	–	–
Ovary weight (mg)	–	–	–	–	47 ± 23	40 ± 14.3
Uterus weight (mg)	–	–	–	–	133 ± 61	184 ± 82

Different letters indicate statistically significant differences (a, b: p < 0.05; A,B: p < 0.02)

### Surgical castration of immuno-deficient nude mice and xenografting experiments

In total, 35 nude mice, aged between 5 and 6 weeks were used (NMRI-*Foxn1nu / Foxn1nu*; Janvier) as recipients for xenografting experiment. The mice were kept at the central animal facility of the medical faculty of the University Münster. Surgical castration of ten nude mice was performed immediately prior to the xenografting procedure. In total, 15 nude mice from intact female (*n* = 5), intact male (*n* = 5) and castrated male (*n* = 5) host groups were used as sham-grafted controls. The remaining 20 nude mice from the three host groups of intact female (*n* = 5), intact male (*n* = 10) and castrated male hosts (*n* = 5) were xenografted with fresh prepubertal marmoset testicular tissue. These mice were kept in the same facility with pellet food and water *ad libitum*. Mice were anesthetized using ketamine xylazine and six fragments per mouse were ectopically xenografted under the back skin of nude mice using Cancer Implant Needles (GI3 Popper and Sons, Staunton, VA, USA) (4). All xenografted and sham-grafted mice were kept in groups of 4–5 mice per cage and were provided pellet food and water. Body weight of all mice was recorded weekly before cage change throughout the 20-week grafting period.

### Serum testosterone analysis

Serum testosterone in serum samples of intact and castrated male mouse hosts was determined using a double-antibody radioimmunoassay protocol (44, 45). An iodinated tracer (testosterone-3-CM-histamine) by the chloramine-T/sodium meta-bisulfite method and an antiserum raised in rabbit against testosterone-3 was used. The detection limit of the assay was 0.68 nmol/l. A serum sample (200 μl) from each mouse was used for testosterone measurements.

### BrdU administration, vaginal smears collection and xenograft retrieval after 20 weeks

After 20-week grafting period, xenografted testicular fragments were retrieved from all host groups. Two hours prior to graft retrieval 100 mg/kg BrdU (5-bromo-2'-deoxyuridine) was injected into each mouse, according to body weight. Vaginal smears were collected from xenografted and shamgrafted female hosts before sacrifice to evaluate their cycling state. Mice were anesthetized using ketamine xylazine and were killed by cardiac puncture. Serum was prepared and stored at −20°C. Each mouse was sacrificed by cardiac puncture. Grafts were collected from the back skin of grafted mice. Ovaries, uterus and vagina were collected from xenografted and sham-grafted female mice. Testes and seminal vesicles were collected from xenografted and sham-grafted male mice. Retrieved marmoset grafts and mice organs were weighed and subsequently fixed in Bouin's solution for further histological analysis.

### Histological analysis

#### PAS staining

Retrieved xenografts and organs from xenografted and sham-grafted male and female mice were fixed in Bouin's solution overnight and were transferred into 70% ethanol (v/v). Pre-graft testicular fragments and retrieved samples were embedded in paraffin and 5 μm thick sections were prepared for histological analysis. Every first and tenth section of each graft as well as sections from pre-graft control fragments were stained with Period acid-Schiff (PAS) / hematoxylin (46) and analyzed for tubule integrity and morphometric characteristics. The analysis of two independent cross-sections at >50 μm distance assured a screening of independent areas during morphometric scoring.

#### Immuno-histochemical analysis of grafts

Immuno-histochemical analysis of graft sections from different host groups was performed to identify different germ cell and somatic cell populations. Sections were deparaffinized and rehydrated followed by antigen retrieval performed in citrate buffer (pH 6). Sections were cooled to room temperature, and after tris-bufferred saline (TBS) wash they were incubated with 3% (v/v) H2O2 (hydrogen peroxidase) for 15 min at room temperature (RT). Non-specific binding sites in the sections were blocked by incubation in the blocking buffer containing 25% chicken/goat serum with 0.5% (w/v) BSA in TBS for 30 min. Sections were incubated overnight at 4°C in different primary antibodies including Melanoma Antigen family A 4, MAGE A4 (received from Prof. G.C. Spagnoli from the University Hospital of Basel Switzerland; dilution 1:20), Boule-like protein, BOLL (166660; Santa cruz; dilution 1:100), ACROSIN (CSF10 Bisonda; dilution 1:200), Proliferating cell nuclear antigen, PCNA (ab92552; Abcam; dilution 1:200), and SRY-BOX9, SOX9 (AB5535; Abcam; dilution 1:50). Control sections were exposed to mouse and rabbit IgG antibodies (Sigma-Aldrich; Hamburg; Germany; dilution 1:1000). Sections were incubated with chicken anti-mouse biotin (Santa Cruz; SC2985; dilution 1:100), and chicken anti-rabbit biotin (Santa Cruz; SC2986; dilution 1:100). After incubation with biotinylated secondary antibodies, sections were incubated with streptavidin horse-raddish peroxidase (HRP) (Sigma-Aldrich; S5512; dilution 1:500) for 45 min. Staining with chromogen 3-3′-diaminobenzidine was performed to observe protein expression patterns. Sections were subsequently counterstained with hematoxylin, dehydrated dehydrated and mounted. Immunofluorescence stainings were performed on graft sections retrieved from male hosts for ACROSIN and CD68 (Supplementary Table 2). Stained sections were visualized using an Olympus BX61 microscope connected to a Retiga 400R camera (Olympus, Melville, NY, USA). Images were captured for analysis using Cellsens imaging software (Olympus, Melville, NY, USA).

#### Estimation of female cyclicity by histological analysis

Fixed uteri and ovaries from sham-grafted and xenografted female mice were sectioned and stained with Period acid-Schiff/hematoxylin to evaluate the reproductive state of the female mice and to record the presence of corpora lutea, endometrium and ongoing folliculogenesis. Giemsa staining of vaginal smears of xenografted and sham-grafted nude mice was performed to estimate cycling status and estrous stages of female mice. Slides were fixed in methanol for 5 min. Giemsa stain was pipetted on top of the smears and slides were dried for 10 min. After distilled water (1x) and methanol (1x) wash, slides were dried under the hood for an hour at RT.

### Semi-quantitative scoring of grafts

**Figure 2:**
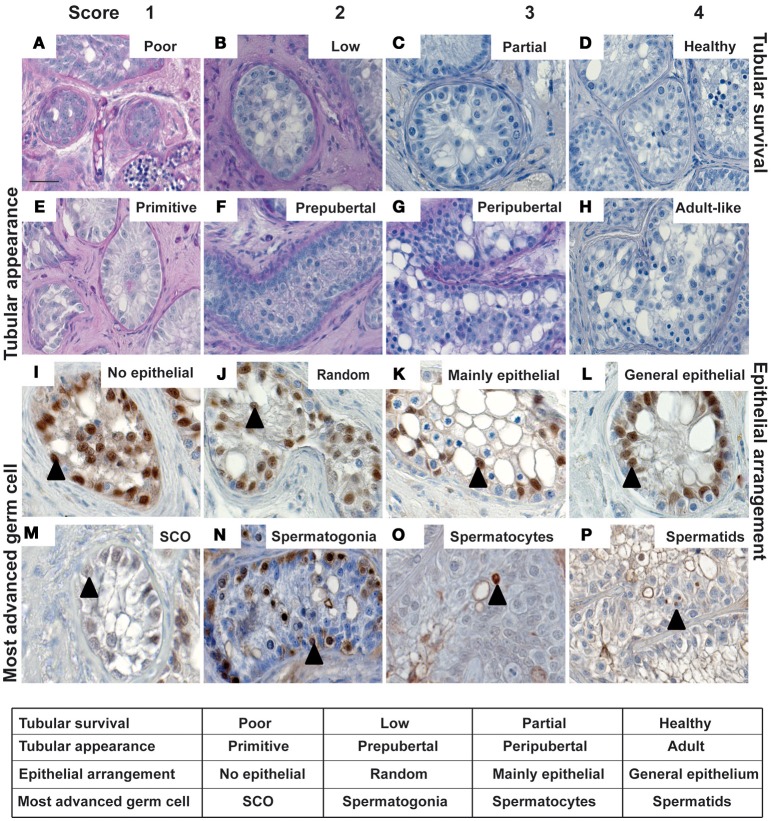
The micrographs represent typical histological features to illustrate the parameters for scoring of morphological characteristics in testicular xenografts. PAS stained sections were evaluated for tubular survival **(A–D)**, tubular appearance **(E–H)**, epithelial pattern of Sertoli cells **(I–L)** and most advanced germ cells **(M–P)**. Blinded scoring of each graft section was performed as depicted in the table. Cellular identity was validated by immuno-histochemical staining. Cells showing intense expression for SOX9, MAGEA4, BOLL, and ACROSIN were identified, indicating the presence of Sertoli cells **(I–M)**, spermatogonia **(N)**, spermatocytes **(O)**, and spermatids **(P)**, respectively. Scale bar 20 μm. Triangles depict representative positive cells for the respective markers.

Morphological characteristics including overall tubular survival, scale of immune infiltration, tubular appearance, epithelial arrangement of Sertoli cells and the most advanced germ cell present in each graft were evaluated by performing blinded scoring of PAS-stained graft sections as demonstrated by representative images in micrographs and scoring scheme in Figure 2. Tubular survival was scored on scale of poor (<10%)–low (10–30%)–partial (30–70%)–healthy (>70%) survival as shown in (Figures 2A–D respectively). Immune infiltration was scored on scale of extreme (>70%)–obvious (30–60%)–initial (10–30%)–no infiltration (>10%) as illustrated by representative micrographs in **Figures 4A**–**D**. As shown in Figures 2E–H, tubular appearance was graded from primitive–prepubertal–peri-pubertal–adult-like appearance of seminiferous tubules. Epithelial arrangement of Sertoli cells was scored from no epithelial–random epithelial arrangement–mainly epithelial–general/normal epithelial as depicted in Figures 2I–L. For the most advanced germ cell type analysis in grafts, sections were scored for grafts with tubules containing Sertoli cell only (SCO)–Spermatogonia–Spermatocytes–Spermatids as demonstrated in Figures 2M–P.

### Semi-quantitative analysis of individual tubules for the most advanced germ cell type present

To evaluate spermatogenic progression, tubule-wise blinded analysis and semi-quantitative scoring of the most advanced germ cell type present in each tubule of all the grafts retrieved from the three host groups was performed. Tubules were categorized into three groups. Tubules containing no germ cells were scored as Sertoli cell only (SCO) tubules, tubules containing pre-meiotic or meiotic/postmeiotic cells as the most advanced germ cell type were scored as 2 independent groups.

### Statistical analysis

Statistical analysis of graft retrieval percentage (among different host groups) was performed using one-way ANOVA followed by Tukey's multiple comparison tests. Paired *T*-tests were used to evaluate statistically significant differences in seminal vesicle weights, graft weights, and mean scores amongst different host groups. Values were considered significantly different if *P* < 0.05. Graph Pad Prism5 (Graph Pad software) was used for all the statistical analysis.

## Results

### Recipient data

Twenty mice were xenografted from which 16 survived during the 20-week grafting period. From all mice, marmoset xenografts were retrieved. Table 2 presents the number of sham-grafted controls and recipients per host group, means of body weight and organ weights (testes, seminal vesicles, ovaries, and uterus). A statistically significant difference was observed in seminal vesicle weight of intact and castrated male hosts.

### Xenograft analysis

One hundred and twenty testicular fragments were xenografted. 71 of 120 grafts were retrieved in total and subjected to histological evaluation revealing that 32 of 71 grafts contained seminiferous tubules. Table 3 depicts absolute and relative numbers of testicular grafts retrieved from different host groups. In addition the calculated retrieval percentages of grafts as well as the weights of grafts are listed. Lowest graft retrieval was recorded in castrated hosts. A significant difference was observed in the graft weight of intact male and female hosts (Table 3).

**Table 3:** Graft retrieval data from intact and castrated immunodeficient nude mouse host.

**Host parameters**	**Intact males**	**Castrated males**	**Intact females**
Grafts retrieved	36/42	13/24	22/30
Graft retrieval (%)	85.71 (A)	54.16 (a,b)	73.30 (B)
Graft weight (mg)	3.2 ± 1 (a)	1.7 ± 0.7	1.2 ± 0.6 (A)

*Different letters indicate statistically significant differences (a, b: p < 0.05; A, B: p < 0.02)*.

### Validation of cellular morphology

Germ and somatic cell populations in the pre-graft controls of all six marmosets were histologically analyzed. Histological characteristics from all the pre-graft control sections demonstrate a prepubertal status (Supplementary Figure 2). Identical histological features were observed in all marmosets. Immuno-histochemical and immunofluorescence staining was thereafter performed to validate the presence of different germ cell populations (pre-meiotic, meiotic, and post-meiotic germ cells) in retrieved xenograft sections as shown in Figures 2N–P and Supplementary Figure 3. Cell population in representative graft sections from all three hosts groups similar to the pre-graft control sections show intense expression of MAGEA4 validating the presence of spermatogonia, as depicted in Figure 2N. Cells in grafts from intact and castrated hosts express BOLL, validating the presence of spermatocytes as illustrated in the representative image in Figure 2O. Cells in grafts recovered from male hosts show strong expression for post-meiotic marker ACROSIN, validating the presence of spermatids as shown in Figure 2P and Supplementary Figure 3. Cells expressing SOX9 were observed in testicular xenografts recovered from all three host groups validating the presence of Sertoli cells in grafts as shown in Figures 2I–L.

### Comparing effect of hosts on morphology and survival of xenografts

**Figure 3:**
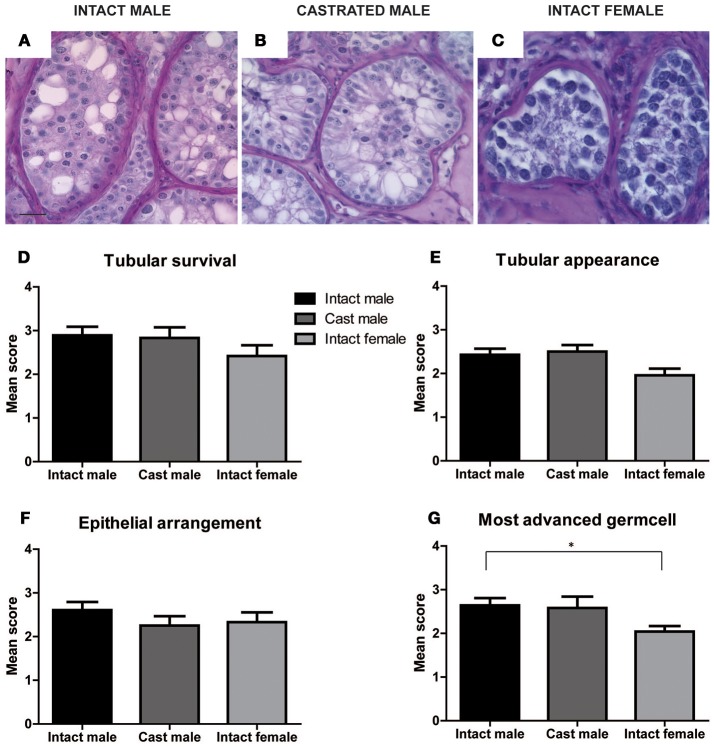
Effect of hosts on tubular survival, tubular appearance, epithelial arrangement and most advanced germ cell was evaluated in xenografts retrieved from intact male, castrated male and intact female hosts by scoring qualitative characteristics of grafts. Representative images of PAS stained xenograft sections from intact male, castrated male and intact female hosts are illustrated in **(A–C)**. Scores for the three host groups for tubular survival **(D)**, tubular appearance **(E)**, epithelial arrangement **(F)**, and most advanced germ cell **(G)** are represented as Mean ± SEM by the bar graphs above. Scale bar 20 μm.

**Figure 4:**
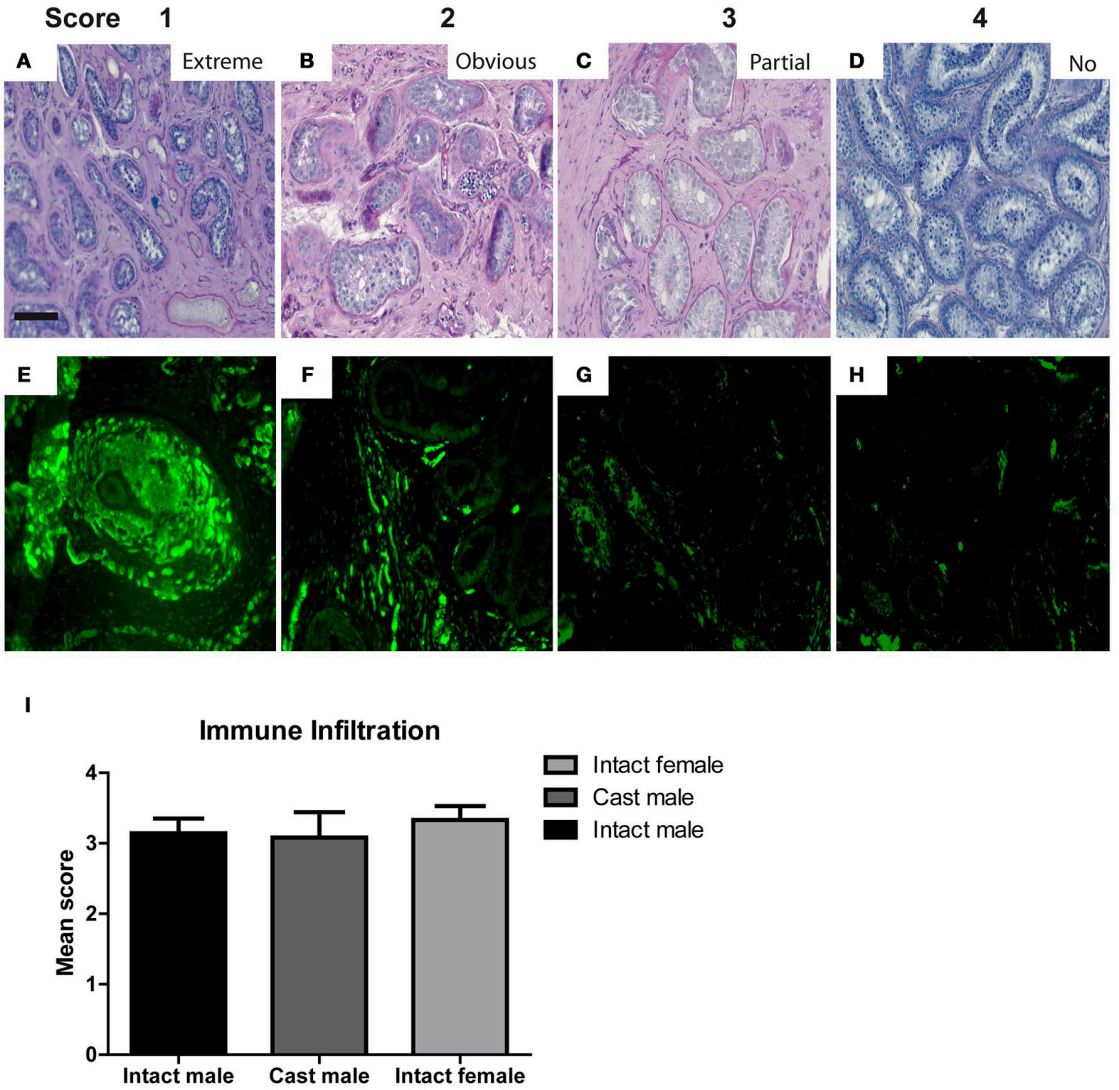
The micrographs represent typical histological features to illustrate the features for scoring of morphological characteristics in testicular xenografts. PAS stained sections were evaluated for Extreme **(A)**, Obvious **(B)**, Partial **(C)**, and No **(D)** immune cell infiltration. Graft sections stained with CD68 antibody show different degrees of host immune cell infiltration **(E–H)**. Scores from the blinded scoring analysis of each graft recovered from three host groups are represented as Mean ± Standard error mean (SEM) in the bar graph above **(I)**. Scale bar 100 μm.

Xenografts retrieved from different host groups were subjected to histological analysis, evaluated morphologically and scored (as shown in Figures 2–**4**) to determine qualitative changes in tubular survival, immune infiltration, tubular appearance, epithelial structure, and spermatogenic state. Overall mean scores (Mean ± SEM) for tubular survival, tubular appearance, epithelial arrangement of Sertoli cells, most advanced germ cell type and immune infiltration are represented in Figures 3, 4, respectively.

As shown in Supplementary Table 1, intact male hosts demonstrate improved (32%) tubular survival compared to female (21%) and castrated hosts (17%). Grafts recovered from intact female hosts were most primitive in tubular appearance (29%) compared to intact (14%) and castrated male host groups (0%). Epithelial arrangement in most of the grafts retrieved from castrated hosts appeared partially random (67%). In contrast to intact (82%) and castrated male hosts (83%), immune cell infiltration was increased in grafts retrieved from the female hosts (92%).

### Comparing the effect of hosts on spermatogenic induction

Most advanced germ cell type analysis of each graft section shows that male hosts contain grafts with the most advanced germ cells up to early spermatids (Figures 3A,B,G, Supplementary Figure 3D,F and Supplementary Table 1). Intact female hosts contain significantly fewer grafts with differentiated germ cells compared to intact males.

Most advanced germ cell analysis of each seminiferous tubule of individual sections (two sections selected 50 μm apart) from each graft was performed to determine the most advanced cell type (SCO, spermatogonia, spermatocytes, spermatids) present. In total 1033 seminiferous tubules from 32 grafts were analyzed from all 3 host groups. 232 tubules were analyzed from 6 grafts of castrated male hosts, 347 tubules were analyzed from 14 grafts retrieved from intact male hosts and 454 tubules were analyzed from 12 grafts retrieved from the female hosts. The highest percentage of grafts recovered from female hosts (62%) contains spermatogonia as the most advanced germ cell type. Both intact and castrated male hosts show improved meiotic (46 and 30%, respectively) and post-meiotic (14 and 20%, respectively) spermatogenic progression compared to female hosts with low meiotic (21%) and no post-meiotic (0%) progression. Grafts retrieved from intact male hosts contain the highest percentage of tubules with post-meiotic germ cells (45.5%). Grafts recovered from female hosts contain the highest percentage of tubules with pre-meiotic germ cells (62.5%). Whereas grafts retrieved from castrated hosts contain highest percentage of tubules with SCOs (32.14%) as demonstrated in Supplementary Table 1.

### Androgen level evaluation in hosts

Testosterone measurements recorded from the serum samples collected from different host groups were heterogeneous and no significant difference in measurement was observed amongst individual host groups. Testosterone levels in sham-grafted control recipients from all three hosts were either low or below detection limits. Testosterone levels in 4 out of 5 castrated recipients were below detection limits, the fifth recipient had very low levels of testosterone. Among intact male recipients, high testosterone levels were recorded in 2 recipients (35.8 and 33.6 nmol/l); while the remaining mice had very low or undetectable testosterone levels. High testosterone levels were recorded in 1 out of 5 intact female recipients (24.8 nmol/l); remaining mice had either very low or undetectable testosterone levels. However, statistically significant difference was observed in freshly retrieved seminal vesicle weights. Seminal vesicle weights represent a widely used bio-indicator of androgen levels in grafting studies as these provide useful and stable readouts of the androgen status compared to the highly fluctuating serum androgen levels in mice (1). Seminal vesicle weight of intact male recipients ranged from 372(±106) to 478(±92) mg, whereas those of castrated recipients was significantly lower, ranging from 17(±2.3) to 20.4(±18) mg (Table 2).

### Effect of grafts on reproductive state of female host

Reproductive state of sham-grafted and xenografted female mice was evaluated by the histological analysis of female reproductive organs (uterus and ovary). Estimation of female cyclicity was performed by Giemsa staining of vaginal smears collected from female mice before sacrifice. This data demonstrates that all the xenografted female mice were at different stages of their estrous cycle similar to the sham-grafted female controls (Supplementary Figure 1). These results are in line with morphological observations of uterine and ovarian sections of the grafted mice, which show the presence of corpora lutea, endometrium, and ongoing folliculogenesis (Supplementary Figure 1).

## Discussion

The xenografting model (ectopic placement of marmoset testicular tissue into nude mice) has been previously used (1, 16). It is also a central strategy in the present study and served as a valid and innovative translational research approach since it allowed the use of limited primate testicular material to generate valid and functional outcomes on spermatogenic induction and testicular development. Xenografting of tissue from a number of species has become a widely used tool to expose immature testicular tissue to different microenvironments (1, 5, 8, 14, 16, 27, 38, 47, 48). In this context, the role of gonadotropins, steroids and age of donor and host have been explored in a number of species (10, 14). In our study it facilitated the analysis of individual testicular grafts (*N* = 120) established from fragments of uniform size derived from 12 immature marmoset testes. Detailed scoring of primate testicular xenografts in combination with effect of hosts of different sex and status were analyzed in our study.

The species-specific role of FSH is still under debate (49). Its role has been described as limited in rodents; however, FSH plays a more active role in primates (50). Xenografting studies are applied as elegant and donor-sparing approaches to explore the testicular FSH response in detail. In man, prepubertal testicular tissue is rarely available to research studies for ethical reasons. However, xenografting studies using fetal human tissue have been performed. As recently reviewed (51) administering exogenous gonadotropin treatment to mouse hosts revealed the stimulation of epithelial maturation and spermatogenic induction in human testicular grafts. First trimester human fetal grafts from hCG treated hosts produced lower testosterone output while second trimester human fetal xenografts produced significantly increased levels of testosterone (52). Leydig cell maturation in grafts from untreated hosts was low whereas obvious Leydig cell differentiation was observed in grafts obtained from hCG treated hosts (52). Similar xenografting studies were performed using infant and juvenile macaque testes as donor material. Confirming the results obtained in human fetal xenografting studies, exogenous gonadotropin treatment resulted in enhanced testicular graft maturation from infantile (3 m, 6 m old) and juvenile (13 m old) rhesus testes (5, 8). No significant influence of human choriogonadotropin (hCG) alone or in combination with hCG and pregnant mare serum gonadotropins (PMSG) was reported on growth of rhesus testicular xenografts (5, 8). However, significantly higher seminal vesicle weights were recorded in exogenously treated xenografted hosts, indicating active Leydig cell function in the grafts (36). Hosts treated with higher dose (10 IU) of hCG demonstrate significantly increased body weights and seminal vesicle weights compared to non-treated and lower dose (1 IU) groups (36). These studies reveal that exogenous treatment of mouse hosts with gonadotropins affects steroid release and gonadal development from primate xenografts and can therefore be used as a preclinical experimental tool (51).

Unlike rodents, old-world primates, or man, the marmoset LH receptor, due to deletion of exon 10 in the LH receptor gene, does not respond to LH but to CG-like hormones (39–43). As mentioned in the introduction, xenografts from marmosets show poor progression of testicular development (1, 16). It was proposed that the mutated LH-receptor in marmosets is causative for the lack of post-meiotic differentiation in marmoset testicular xenografts. Since mice do not express CG, the mouse host does not stimulate the marmoset LH-responsive testicular Leydig cells and therefore the testosterone release is at baseline level. However an intact endocrine milieu is sufficient for stimulating full spermatogenesis in autologous grafts at the scrotal site in marmosets (37). Even co-grafting with hamster tissue did not overcome the blockade of development (16). In contrast to macaques and man, the marmoset tissue offers a peculiar endocrine situation providing options for delineating the LH and FSH actions since LH action is diminished in the mouse host. Before performing studies with substitution of exogenous hormones to the host we were interested to learn about the effects of different host environments on marmoset xenograft development. We showed that the endocrine status of the three different hosts had different effects on graft survival and spermatogenic progression. Taking into account the dysfunctional LH-testosterone axis in marmosets our selection of hosts created three specific hormonal microenvironments for the grafts. The intact recipients generated an endogenous androgen exposure with normal mouse FSH serum levels. The castrated hosts exposed the grafts to high mouse FSH serum levels under complete absence of endogenous testosterone. The endocrine milieu of female hosts consists of variable levels of serum estrogens (as the females continued cycling) in combination with very low testosterone under normal mouse FSH serum levels. Observing unaffected cyclicity in intact female hosts leads us to assume that intact male hosts were not affected by the release of androgens from the xenografts. Comparable levels of seminal vesicle weights in sham-grafted castrated recipients substantiated that the androgen release from marmoset grafts was low as had been shown in previous xenografting studies using marmosets. All these findings confirm dysfunctionality of the LH-receptor in marmoset testicular tissue. We assume that our data will help to design appropriate experimental schemes to explore more specifically the role of FSH in primates.

Nude mice are historically widely used models to explore the developmental aspects of tumors. They are also used for studying the growth and differentiation of immature tissues. Over many years they became standardized models for (xeno-)grafting studies (14). Previous reports strongly suggest that host effects between individual mice are negligible (1, 14, 16, 53–55). Testicular xenografting studies investigating the effect of host castration (5), male or female host (48), co-grafting (16), single and group-housing (54), younger and older host recipients (55) report identical outcome and comparable effects on individual mice from experimental groups (14). Even studies investigating the effect of irradiation in testicular xenografts at a cellular level show similar effects in groups of hosts. Similar doses of irradiation revealed identical deleterious effects on germ and somatic cell populations in the grafts of each group (53). Likewise in the current study, body weights and organ weights were comparable between individual hosts in specific host groups and in the expected normal range. There was no evidence for a high variability due to individual hosts. Thus, neither in the current study, nor in previous xenografting studies using testis tissue, a “host effect” of individual nude mice on testis grafts could be detected.

Grafting in intact hosts (male and female), lead to recovery rates of grafts much better than reported in castrate recipients (current manuscript, 1, 16]. We consider therefore the intact host environment to be effective for marmoset testis tissue survival and development. We assume that the presence of steroids irrespective of being androgens or estrogens plays a major role for survival. In the absence of steroids in castrates the survival of xenografts was rather poor. Surprisingly the intact hosts showed obvious differences in graft development with male hosts being superior to females in promoting spermatogenic induction. We can only speculate that either the absence of androgens or the fluctuations in FSH created this difference. In future studies exogenous hormone delivery using various host conditions may be an elegant strategy to explore the actions of hormones during spermatogenic induction.

Grafts retrieved from intact male hosts show most advanced tubular survival, seminiferous epithelial arrangement, tubular, and luminal enlargement, least immune cell infiltration and most prominent progression of spermatogenesis. Indeed all previous studies on xenografting of immature monkey tissue used castrated male hosts. Castration was considered advantageous since the loss of endogenous testes creates a microenvironment with high gonadotropic drive considered to stimulate the maturation of grafted tissue. Another advantage of using castrated hosts is that any testosterone action reflects androgen action of the grafts. This might also be considered valid for our study as we cannot separate donor and host steroids in intact recipients. Since mice show extreme variation in serum steroid levels, the size of the recipient's seminal vesicles was selected as the parameter for the release of androgens and therefore presented an easy readout of the activity along the endocrine axis (3, 4).

Grafts from all three hosts showed intense expression for MAGEA4. The staining expression patterns were similar to testes from age-matched marmoset controls and indicate the presence of spermatogonia in all host conditions. Unlike previous marmoset ectopic grafting studies reporting germ cell maturation up to the stage of early spermatocytes (1, 4, 16, 36), ectopic grafts from male hosts expressed BOLL (spermatocyte marker) and ACROSIN (spermatid marker) revealing the presence of spermatocytes and occasionally spermatids in grafts from both, intact and castrated male mice. Post-meiotic development stops at the stage of early spermatids. Interestingly female hosts did not support post-meiotic progression. The striking host-dependent effects were substantiated by analysis of the most advanced germ cell types showing significant differences in the degree of post-meiotic germ cell differentiation between intact male and female hosts. This observation is in agreement with a study on newborn piglet testicular tissue revealing that poor post-meiotic differentiation is observed in female hosts compared to males (25). In mice a syngeneic testicular grafting study showed efficient graft survival and good post-meiotic differentiation in female recipients (48). Female hosts seem to be good recipients for xenografts in terms of graft survival. However comparison with marmosets is difficult as in all other species the endogenous mouse LH induces production of androgens in the grafts leading to intra-testicular rise of male steroids. For instance, very high androgen levels have been recorded in pig xenografts (7). Species-specific differences may therefore lead to highly variable endocrine conditions in xenografted testis tissue. The marmoset may be an extreme model with no LH action present and therefore highly reduced testosterone stimulation. This observation is in agreement with data from previous marmoset xenografting studies achieving a low androgen status in castrated hosts irrespective of the age of donor tissue (1, 3, and 7 months of age) (16).

This is the first study comparing the effects of the host environment via xenografting of marmoset immature testes into intact and castrated male and female mouse immuno-deficient hosts. We observed changes of xenograft survival, testicular maturation and spermatogenic induction. Intact male hosts support most efficiently the survival of as well as the spermatogenic induction and progression in prepubertal marmoset testicular xenografts. The effects were put into context with the peculiar regulation of androgens in marmosets. Current findings hold clinical relevance signifying that marmoset monkeys could potentially be employed as a preclinical model to understand the hormonal control of spermatogenesis.

## Author contributions

SwS performed the animal study and data analysis, compiled the manuscript. RS-K assisted with animal studies, determined hormone levels, assisted with study design. StS acted as PI during study planning and performance, and assisted with paper writing.

### Conflict of interest statement

The authors declare that the research was conducted in the absence of any commercial or financial relationships that could be construed as a potential conflict of interest.

## References

[1] Schlatt S Kim SS Gosden R. Spermatogenesis and steroidogenesis in mouse, hamster and monkey testicular tissue after cryopreservation and heterotopic grafting to castrated hosts. Reproduction (2002) 124:339–46. 10.1530/rep.0.1240339 12201807

[2] Schlatt S Westernströer B Gassei K Ehmcke J. Donor-host involvement in immature rat testis xenografting into nude mouse hosts. Biol Reprod. (2010) 82:888–95. 10.1095/biolreprod.109.082073 20107205 PMC2857632

[3] Schlatt S Honaramooz A Boiani M Schöler HR Dobrinski I. Progeny from sperm obtained after ectopic grafting of neonatal mouse testes. Biol Reprod. (2003) 68:2331–5. 10.1095/biolreprod.102.014894 12606381

[4] Honaramooz A Snedaker A Boiani M Schöler HR Dobrinski I Schlatt S. Sperm from neonatal mammalian testes grafted in mice. Nature (2002) 418:778–81. 10.1038/nature00918 12181567

[5] Honaramooz A Li MW Penedo MC Meyers S Dobrinski I. Accelerated maturation of Primate Testis by Xenografting into Mice. Biol Reprod. (2004) 70:1500–3. 10.1095/biolreprod.103.025536 14736818

[6] Oatley JM Avila DM Reeves JJ McLean DJ. Spermatogenesis and Germ cell transgene expression in xenografted bovine testicular tissue. Biol Reprod. (2004) 71:494–501. 10.1095/biolreprod.104.027953 15070832

[7] Arregui L Rathi R Zeng W Honaramooz A Gomendio M Roldan ERS. Xenografting of adult mammalian testis tissue. Anim Reprod Sci. (2008) 106:65–76. 10.1016/j.anireprosci.2007.03.026 17512146 PMC2386512

[8] Rathi R Zeng W Megee S Conley A Meyers S Dobrinski I. Maturation of testicular tissue from infant monkeys after xenografting into mice. Endocrinology (2008) 149:5288–96. 10.1210/en.2008-0311 18566126 PMC2582907

[9] Liu Z Nie YH Zhang CC Cai YJ Wang Y Lu HP. Generation of macaques with sperm derived from juvenile monkey testicular xenografts. Nature (2015) 26:139–42. 10.1038/cr.2015.112 26369429 PMC4816125

[10] Rodriguez Sosa JR Dobrinski I. Recent developments in testis tissue xenografting. Reproduction (2009) 138:187–94. 10.1530/REP-09-0012 19372227

[11] Schlatt S Ehmcke J Jahnukainen K. Testicular stem cells for fertility preservation: Preclinical studies on male germ cell transplantation and testicular grafting. Pediatr Blood Cancer (2009) 53:274–80. 10.1002/pbc.22002 19415740

[12] Sato Y Nozawa S Yoshiike M Arai M Sasaki C Iwamoto T. Xenografting of testicular tissue from an infant human donor results in accelerated testicular maturation. Hum Reprod. (2010) 25:1113–22. 10.1093/humrep/deq001 20172867

[13] Wyns C Curaba M Vanabelle B Langendonckt AV Donnez J. Options for fertility preservation in prepubertal boys. Hum Reprod Update (2010) 16:312–8. 10.1093/humupd/dmp054 20047952

[14] Arregui L Dobrinski I. Xenografting of testicular tissue pieces: 12 years of an *in vivo* spermatogenesis system. Reproduction (2014) 148:R71–84. 10.1530/REP-14-0249 25150043 PMC4277018

[15] Picton HM Wyns C Anderson RA Goossens E Jahnukainen K Kliesch S. A European perspective on testicular tissue cryopreservation for fertility preservation in prepubertal and adolescent boys. Hum Reprod. (2015) 30:2463–75. 10.1093/humrep/dev190 26358785

[16] Wistuba J Mundry M Luetjens CM Schlatt S Co-grafting of hamster *(Phodopus- Sungorus)* and marmoset (*Callithrix jacchus*) testicular tissues into nude mice does not overcome blockade of early spermatogenic differentiation in primate grafts. Biol Reprod. (2004) 71:2087–91. 10.1095/biolreprod.104.033431 15317690

[17] Jahnukainen K Ehmcke J Hergenrother SD Schlatt S. Effect of cold storage and cryopreservation of immature non-human primate testicular tissue on spermatogonial stem cell potential in xenografts. Hum Reprod. (2007) 22:1060–7. 10.1093/humrep/del/471 17166865

[18] Jahnukainen K Ehmcke J Nurmio M Schlatt S. Autologous ectopic grafting of cryopreserved testicular tissue preserves the fertility of prepubescent monkeys that receive sterilizing cytotoxic therapy. Cancer Res. (2012) 72:5174–8. 10.1158/0008-5472.CAN-12-1317 22902414 PMC3971428

[19] Geens M Block GD Goossens E Frederickx V Steirteghem AV Tournaye H. Spermatogonial survival after grafting human testicular tissue to immunodeficient mice. Hum Reprod. (2006) 21:390–396. 10.1093/humrep/dei412 16311289

[20] Schlatt S Honaramooz A Ehmcke J Goebell PJ Rübben H Dhir R. Limited survival of adult human testicular tissue as ectopic xenograft. Hum Reprod. (2006) 21:384–9. 10.1093/humrep/dei352 16239313 PMC1361612

[21] Yu J Cai ZM Wan HJ Zhang FT Ye J Fang JZ. Development of neonatal mouse and fetal human testicular tissue as ectopic grafts in immunodeficient mice. Asian J Androl. (2006) 8:393–403. 10.1111/j.1745-7262.2006.00189.x 16763714

[22] Wyns C Van Langendonckt A Wese FX Donnez J Curaba M. Long-term spermatogonial survival in cryopreserved and xenografted immature human testicular tissue. Hum Reprod. (2008) 23:2402–14. 10.1093/humrep/den272 18664476

[23] Shinohara T Inoue K Ogonuki N Kanatsu-Shinohara M Miki H Nakata K. Birth of offspring following transplantation of cryopreserved immature testicular pieces and *in-vitro* microinsemination. Hum Reprod. (2002) 17:3039–45. 10.1093/humrep/17.12.3039 12456600

[24] Huan S Sartini BL Parks JE Spermatogenesis in testis xenografts grafted from pre-pubertal Holstein bulls is re-established by stem cell or early spermatogonia. Anim Reprod Sci. (2006) 103:1–12. 10.1016/j.anireprosci.2006.11.018 17188436

[25] Abbasi S Honaramooz A. Effects of recipient mouse strain, sex and gonadal status on the outcome of testis tissue xenografting. Reprod Fertility Dev. (2010) 22:1279–86. 10.1071/RD10084 20883654

[26] Reddy N Mahla RS Thathi R Suman SK Jose J Goel S. Gonadal status of male recipient mice influences germ cell development in immature buffalo testis tissue xenograft. Reproduction (2012) 143:59–69. 10.1530/REP-11-0286 22046056

[27] Schlatt S Gassei K Westernströer B Ehmcke J. Modulating testicular mass in xenografting: a model to explore testis development and endocrine function. Endocrinology (2010) 151:4018–23. 10.1210/en.2010-0415 20555023 PMC2940526

[28] McKinnell C Mitchell RT Morris K Anderson RA Kelnar CJH Wallace WH. Perinatal germ cell development and differentiation in the male marmoset (*Callithrix jacchus*): similarities with the human and differences from the rat. Hum Reprod. (2013) 28:886–96. 10.1093/humrep/des465 23321215 PMC3600838

[29] Mitchell RT Cowan G Morris KD Anderson RA Fraser HM Mckenzie KJ. Germ cell differentiation in the marmoset (*Callithrix jacchus*) fetal and neonatal life closely parallels that in the human. Hum Reprod. (2008) 23:2755–65. 10.1093/humrep/den295 18694875 PMC2583943

[30] Kelnar CJ McKinnell C Walker M Morris KD Wallace WH Saunders PT. Testicular changes during infantile ‘quiescence’ in the marmoset and their gonadotrophin dependence: a model for investigating susceptibility of the prepubertal human testis to cancer therapy? Hum Reprod. (2002) 17:1367–78. 10.1093/humrep/17.5.1367 11980767

[31] Irfan S Wistuba J Ehmcke J Shahab M Schlatt S Pubertal and testicular development in the common marmoset (*Callithrix jacchus*) shows high individual variation. Prim Biol. (2015) 2:1–8. 10.5194/pb-2-1-2015

[32] Sharpe RM Fraser HM Brougham MF McKinnell C Morris KD Kelnar CJ. Role of the neonatal period of pituitary-testicular activity in germ cell proliferation and differentiation in the primate testis. Hum Reprod. (2003) 18:2110–7. 10.1093/humrep/deg413 14507830

[33] Li L-H Donald JM Golub MS. Testicular development, structure, function and regulation in common marmoset. Birth Defects Res. (2005) 74:450–69. 10.1002/bdrb.20057 16193499

[34] McKinnell C Mitchell RT Walker M Morris K Kelnar CJ Wallace WH. Effect of fetal or neonatal exposure to monobutyl phthalate (MBP) on testicular development and function in the marmoset. Hum Reprod. (2009) 24:2244–54. 10.1093/humrep/dep200 19491204 PMC2727403

[35] Mitchell RT Saunders PT Sharpe RM Kelnar CJ Wallace WH. Male fertility and strategies for fertility preservation following childhood cancer treatment. Endocr Dev. (2009) 15:101–34. 10.1159/000207612 19293606

[36] Wistuba J Luetjens CM Wesselmann R Nieschlag E Simoni M Schlatt S. Meiosis in autologous ectopic transplants of immature testicular tissue grafted to *Callithrix jacchus*. Biol Reprod. (2006) 74:706–13. 10.1095/biolreprod.105.048793 16371588

[37] Luetjens CM Stukenborg JB Nieschlag E Simoni M Wistuba J Complete spermatogenesis in orthotopic but not in ectopic transplants of autologously grafted marmoset testicular tissue. Endocrinology (2008) 149:1736–47. 10.1210/en.2007-1325 18174281

[38] Ehmcke J Gassei K Westernströer B Schlatt S Immature rhesus monkey (Macaca mulatta) testis xenografts show increased growth, but not enhanced seminiferous differentiation, under human chorionic gonadotropin treatment of nude mouse recipients. Int J Androl. (2011) 34:e459–67. 10.1111/j.1365-2605.2011.01179.x 21651576

[39] Gromoll J Wistuba J Terwort N Godmann M Muller T Simoni M. A new subclass of the lutenizing hormone / chorionic gonadotropin receptor lacking exon 10 messenger RNA in the New world monkey (Platyrrhini) lineage. Biol. Reprod. (2003) 69:75–80. 10.1095/biolreprod.102.014902 12606382

[40] Muller T Simoni M Pekel E Luetjens CM Chandolia R Amato F. Chorionic Gonadotrophin beta subunit mRNA but not luteinizing hormone beta subunit mRNA is expressed in the pituitary of the common marmoset (*Callithrix jacchus*). J Mol Endocrinol. (2004) 32:115–28. 10.1677/jme.0.0320115 14765996

[41] Müller T Gromoll J Simula AP Norman R Sandhowe-Klaverkamp R Simoni M. The carboxyterminal peptide of chorionic gonadotropin facilitates activation of the marmoset LH receptor. Exp Clin Endocrinol Diabetes (2009) 112:574–9. 10.1055/s-2004-830409 15578332

[42] Tannenbaum PL Schultz-Darken NJ Woller MJ Abbott DH. Gonadotrophin-releasing hormone (GnRH) release in marmosets II: pulsatile release of GnRH and pituitary gonadotrophin in adult females. J Neuroendocrinol. (2007) 19:354–63. 10.1111/j.1365-2826.2007.01535.x 17425610

[43] Henke A Luetjens CM Simoni M Gromoll J. Chorionic Gonadotropin β-subunit gene expression in the Marmoset pituitary is controlled by steroidogenic factor 1, early growth response protein 1, and pituitary homeobox factor 1. Endocrinology (2007) 148:6062–72. 10.1210/en.2007-0825 17872365

[44] Chandolia RK Weinbauer GF Simoni M Behre HM Nieschlag E. Comparative effects of chronic administration of the non-steroidal antiandrogens flutamide and casodex on the reproductive system of the adult male rat. Acta Endocrinologica (1991) 125:547–55. 10.1530/acta.0.1250547 1759544

[45] Chandolia RK Luetjens CM Wistuba J Yeung C-H Nieschlag E Simoni M. Changes in endocrine profile and reproductive organs during puberty in the male marmoset monkey (Callithrix jacchus). Reproduction (2006) 132:355–63. 10.1530/rep.1.01186 16885543

[46] Brinkworth MH Weinbauer GF Schlatt S Nieschlag E. Identification of male germ cells undergoing apoptosis in adult rats. J Reprod Fertility (1995) 105:25–33. 10.1530/jrf.0.1050025 7490711

[47] Spade DJ Hall SJ Saffarini CM Huse SM McDonnell EV Boekelheide K. Differential response to abiraterone acetate and di-n-butyl phthalate in an androgen-sensitive human fetal testis xenograft bioassay. Toxicol Sci. (2014) 138:148–60. 10.1093/toxsci/kft266 24284787 PMC3930360

[48] Ma P Ge Y Wang S Ma J Xue S Han D. Spermatogenesis following syngeneic testicular transplantation in Balb/c mice. Reproduction (2004) 128:163–70. 10.1530/rep.1.00165 15280555

[49] Schlatt S Ehmcke J. Regulation of spermatogenesis: an evolutionary biologist's perspective. Semin Cell Dev Biol. (2014) 29:2–16. 10.1016/j.semcdb.2014.03.007 24685618

[50] Plant TM Marshall GR. The functional significance of FSH in spermatogenesis and the control of its secretion in male primates. Endocr Rev. (2001) 22:764–86. 10.1210/edrv.22.6.0446 11739331

[51] Hutka M Smith LB Mitchell RT. Xenotransplantation as a model for human testicular development. Differentiation (2017) 97:44–53. 10.1016/j.diff.2017.09.001 28946057

[52] Mitchell RT Saunders PTK Childs AJ Cassidy-Kojima C Anderson RA Wallace WHB. Xenografting of human fetal testis tissue: a new approach to study fetal testis development and germ cell differentiation. Hum Reprod. (2010) 25:2405–14. 10.1093/humrep/deq183 20683063 PMC2939754

[53] Tröndle I Westernströer B Wistuba J Terwort N Schlatt S Neuhaus N. Irradiation affects germ and somatic cells in prepubertal monkey testis xenografts. Mol Hum Reprod. (2017) 23:141–54. 10.1093/molehr/gax003 28130393

[54] Arregui L Rathi R Megee SO Honaramooz A Gomendio M Roldan E. Xenografting of sheep testis tissue and isolated cells as a model for preservation of genetic material from endangered ungulates. Reproduction (2008) 136:85–93. 10.1530/REP-07-0433 18390693

[55] Ehmcke J Gassei K Schlatt S Ectopic testicular xenografts from newborn hamsters (*Phodopus sungorus*) show better spermatogenic activity in aged compared with young recipients. J Exp Zool Part A (2008) 309A:278 10.1002/jez.459 18412097

