# Supplementary material for: Differentiation of Testis Xenografts in the Prepubertal Marmoset Depends on the Sex and Status of the Mouse Host
Source: Front Endocrinol (Lausanne). 2018 Aug 29;9:467. doi: 10.3389/fendo.2018.00467 (PMC6123353; doi:10.3389/fendo.2018.00467)
Supplement: Supplementary file 1 [file Data_Sheet_1.doc]

Supplementary Material

# Differentiation of testis xenografts in the prepubertal marmoset depends on the sex and status of the mouse host

Swati Sharma, Reinhild Sandhowe-Klaverkamp, Stefan Schlatt*

*** Correspondence:** Corresponding Author: [stefan.schlatt@ukmuenster.de](mailto:stefan.schlatt@ukmuenster.de)

# Supplementary Data

**1.1 Supplementary Figures**

**
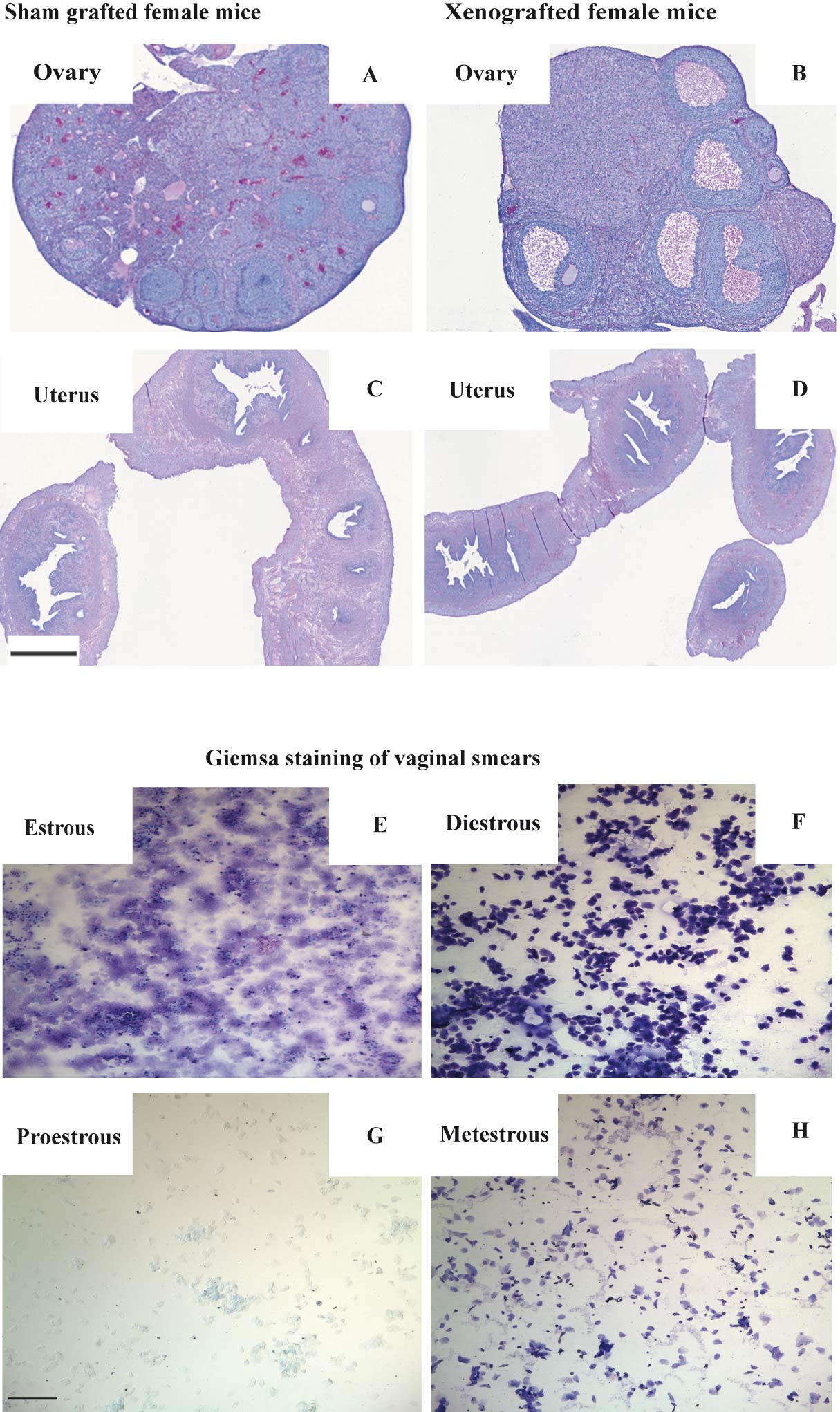
**

**Supplementary Figure 1.** Micrographs depict PAS stained cross-sections of sham-grafted and xenografted female mice ovary (A, B) and uterus (C, D) and Giemsa stained vaginal smears of xenografted female mice (E, F, G, H). PAS staining of female mice organs demonstrates active reproductive cyclicity of the sham-grafted and xenografted female mice. Scale bar (1 mm), 60x. Giemsa staining of vaginal smears collected from xenografted mice confirms maintenance of estrous cycles, i.e Estrous (E), Diestrous (F), Proestrous (G) and Metestrous (H). Scale bar (20 µm), 20X.

**
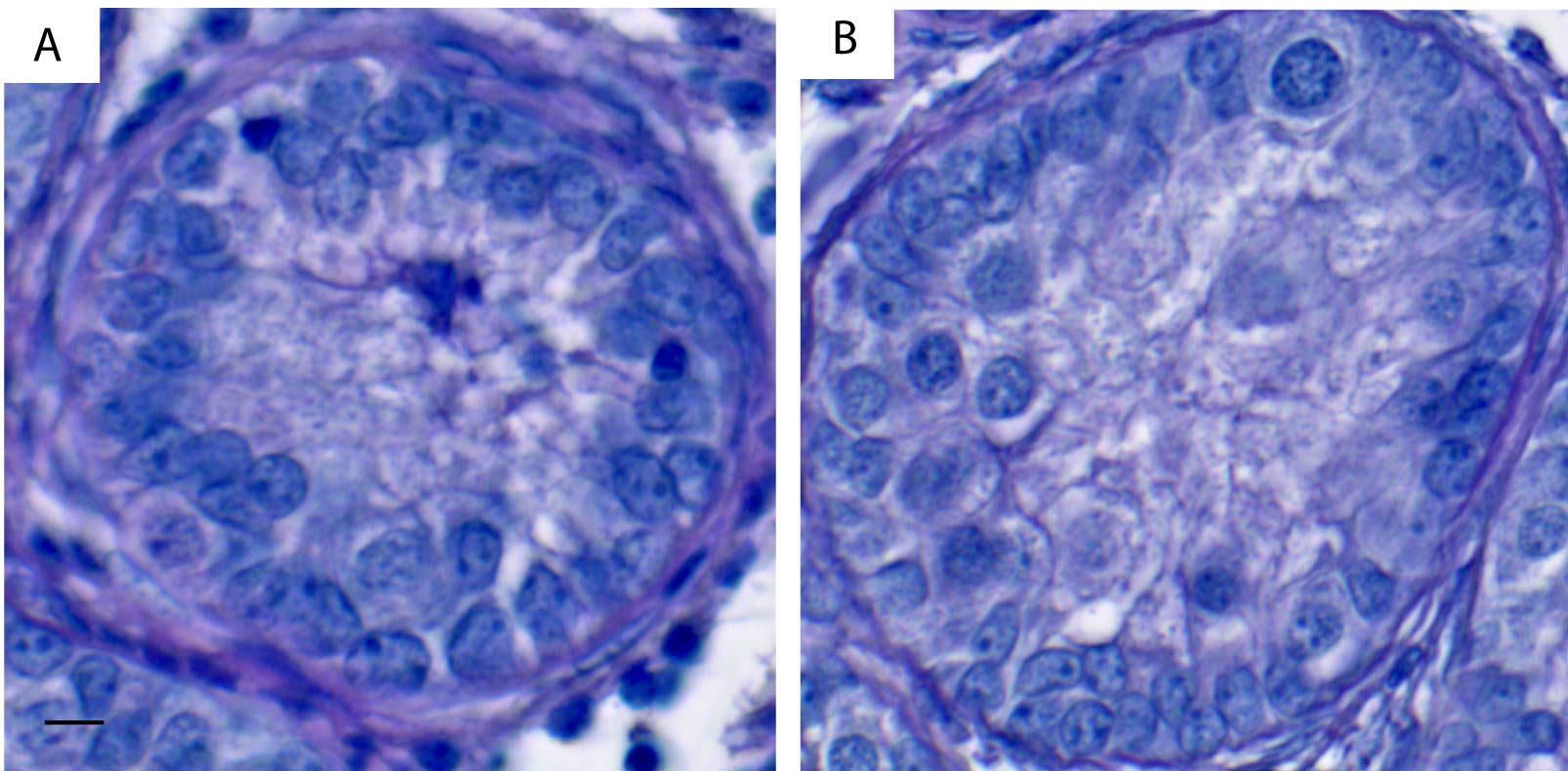
**

**Supplementary Figure 2.** Micrographs depict a prepubertal status of PAS stained testicular cross-sections of pre-graft controls (A, B) from two different marmoset donors (MC945, MC947). Immature Sertoli cells and spermatogonia represent the only cell types in the cords. Scale bar (10 µm).

**
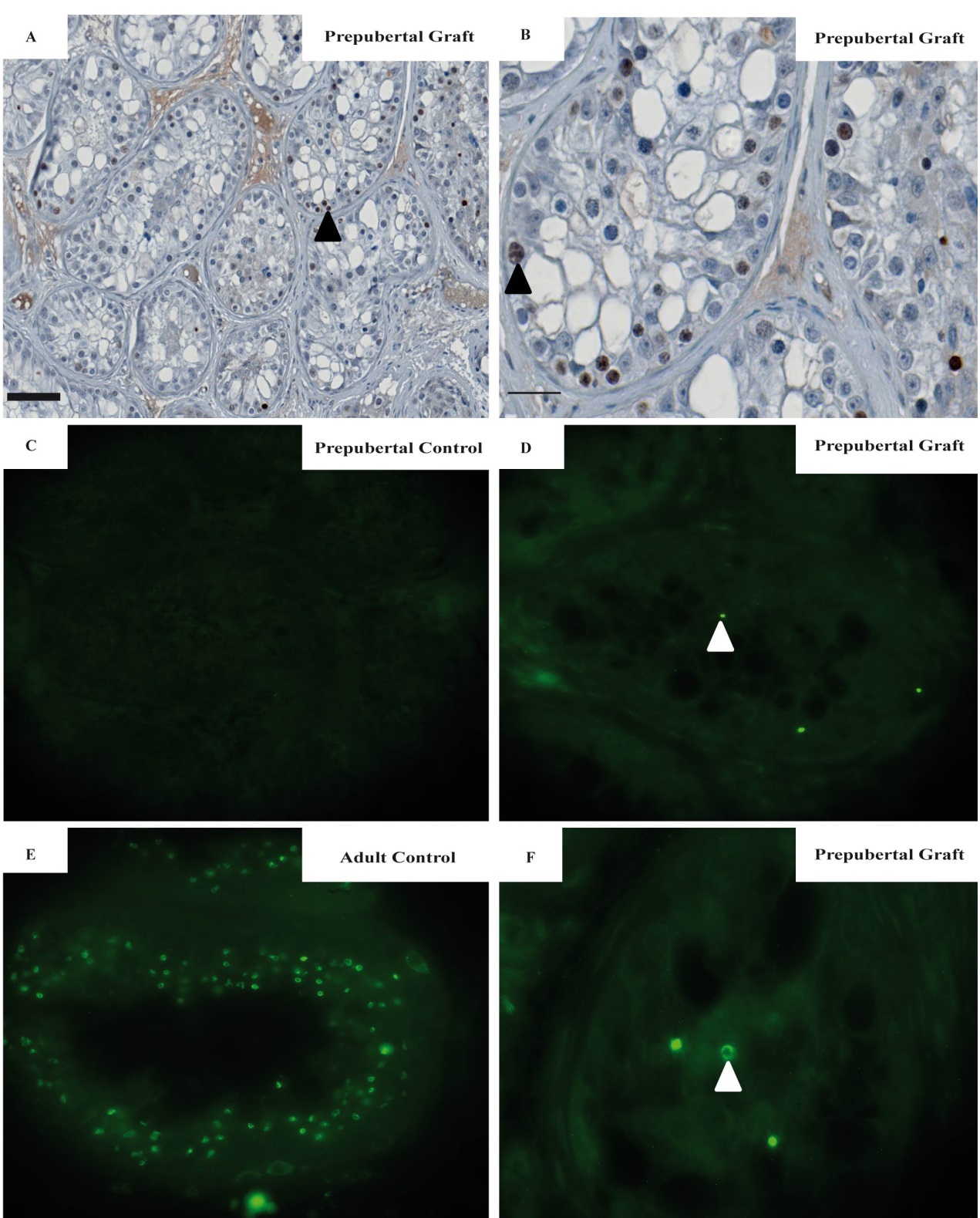
**

**Supplementary Figure 3.** Micrographs illustrate cells showing proliferation and post-meiotic differentiation in prepubertal marmoset testicular xenograft sections retrieved from intact male host. Positively stained cells for cell proliferation marker PCNA in prepubertal marmoset testicular sections are illustrated (A and B). Micrographs show immunofluorescence staining in prepubertal (C) and adult (E) marmoset testicular control and xenograft sections (D and F) retrieved from intact male hosts for post-meiotic spermatid marker ACROSIN. Adult marmoset testicular control sections (E) and prepubertal marmoset testicular xenograft sections (D and F) retrieved from intact male hosts show ACROSIN positive expression at immunofluorescence level. No staining was observed in prepubertal marmoset testicular control section (A). Scale bar 100 µm and 20 µm.

**1.2 Supplementary Tables**

**Supplementary Table 1(A).** Percentage of Grafts (%) representing different scores for three hosts. Two sections (50µm apart) from each graft were analyzed for tubular survival, tubular appearance, epithelial arrangement, immune infiltration and most advanced germ cells present in grafts. Number of grafts analyzed from each host group were as follows: Intact male host (14 grafts:28 sections), Castrated male host (6 grafts:12 sections), Intact female host (12 grafts:24 sections). **Supplementary Table 1(B).** Percentage of tubules (%) from three hosts containing most advanced germ cell. Total number of tubules analyzed from each host group was as follows: Intact male host (347 tubules), Castrated male host (232 tubules) and Intact female host (454 tubules).

|  | **Graft (%)** | **Intact male** | **Castrated male** | **Intact female** |
| --- | --- | --- | --- | --- |
| **Tubular survival** | **Poor** | 15 | 8 | 37 |
|  | **Low** | 14 | 17 | 4 |
|  | **Partial** | 39 | 58 | 38 |
|  | **Healthy** | 32 | 17 | 21 |
| **Tubular appearance** | **Primitive** | 14 | 0 | 29 |
|  | **Prepubertal** | 36 | 50 | 25 |
|  | **Peripubertal** | 50 | 50 | 46 |
|  | **Adult** | 0 | 0 | 0 |
| **Epithelial arrangement** | **No epithelial** | 18 | 8 | 29 |
|  | **Partially random** | 21 | 67 | 25 |
|  | **Mainly epithelial** | 43 | 17 | 29 |
|  | **General epithelial** | 18 | 8 | 17 |
| **Immune infiltration** | **No** | 18 | 17 | 8 |
|  | **Initial** | 0 | 17 | 8 |
|  | **Obvious** | 32 | 8 | 25 |
|  | **Extreme** | 50 | 58 | 59 |
| **Most advanced germ cell in grafts** | **SCO** | 11 | 10 | 17 |
|  | **Spermatogonia** | 29 | 40 | 62 |
|  | **Spermatocytes** | 46 | 30 | 21 |
|  | **Spermatids** | 14 | 20 | 0 |

|  | **Tubule (%)** | **Intact male** | **Castrated male** | **Intact female** |
| --- | --- | --- | --- | --- |
| **Most advanced germ cell in tubules** | **SCO** | 10.32 | 32.14 | 16.77 |
|  | **Premeiotic** | 44.16 | 37.92 | 62.47 |
|  | **≥ Meiotic** | 45.50 | 29.92 | 20.73 |

**Supplementary Table 2. Details of antibodies used for immunohistological analysis.**

| **Primary antibodies** | **Host** | **Antibody dilution** | **Catalogue number** | **Brand** |
| --- | --- | --- | --- | --- |
| MAGEA4 | Mouse | 1:50 | - | (Provided by Prof. Spagnoli*) |
| BOLL | Mouse | 1:100 | 166660 | Santa cruz Biotech |
| ACROSIN | Mouse | 1:300 | CSF10 | Biosonda |
| PCNA | rabbit | 1:200 | Ab92552 | Abcam |
| SOX9 | Rabbit | 1:50 | AB5535 | Merck |
| CD68 | Mouse | 1:50 | 11-0689-42 | Thermofischer Scientific |

| **Secondary and tertiary antibodies** | **Antibody dilution** | **Catalogue number** | **Brand** |
| --- | --- | --- | --- |
| Chicken-anti-mouse biotinylated | 1:100 | SC2985 | Santa cruz Biotech |
| Goat-anti-rabbit HRP | 1:100 | A6154 | Sigma |
| Goat-anti-mouse 488 | 1:100 | A11001 | Invitrogen |
| Streptavidin-HRP | 1:500 | S5512 | Sigma |

*Provided by Prof. G.C. Spagnoli from the University Hospital of Basel, Switzerland
